# Supplementary material for: Vitamin D Modified DSS-Induced Colitis in Mice via STING Signaling Pathway
Source: Biology (Basel). 2025 Jun 18;14(6):715. doi: 10.3390/biology14060715 (PMC12190092; doi:10.3390/biology14060715)
Supplement: Supplementary file 1 [file biology-14-00715-s001.zip › Supplementary Materials S1: 16s-report of gut microbiota/Groups/Lefse_Analysis/out_formant.pdf]

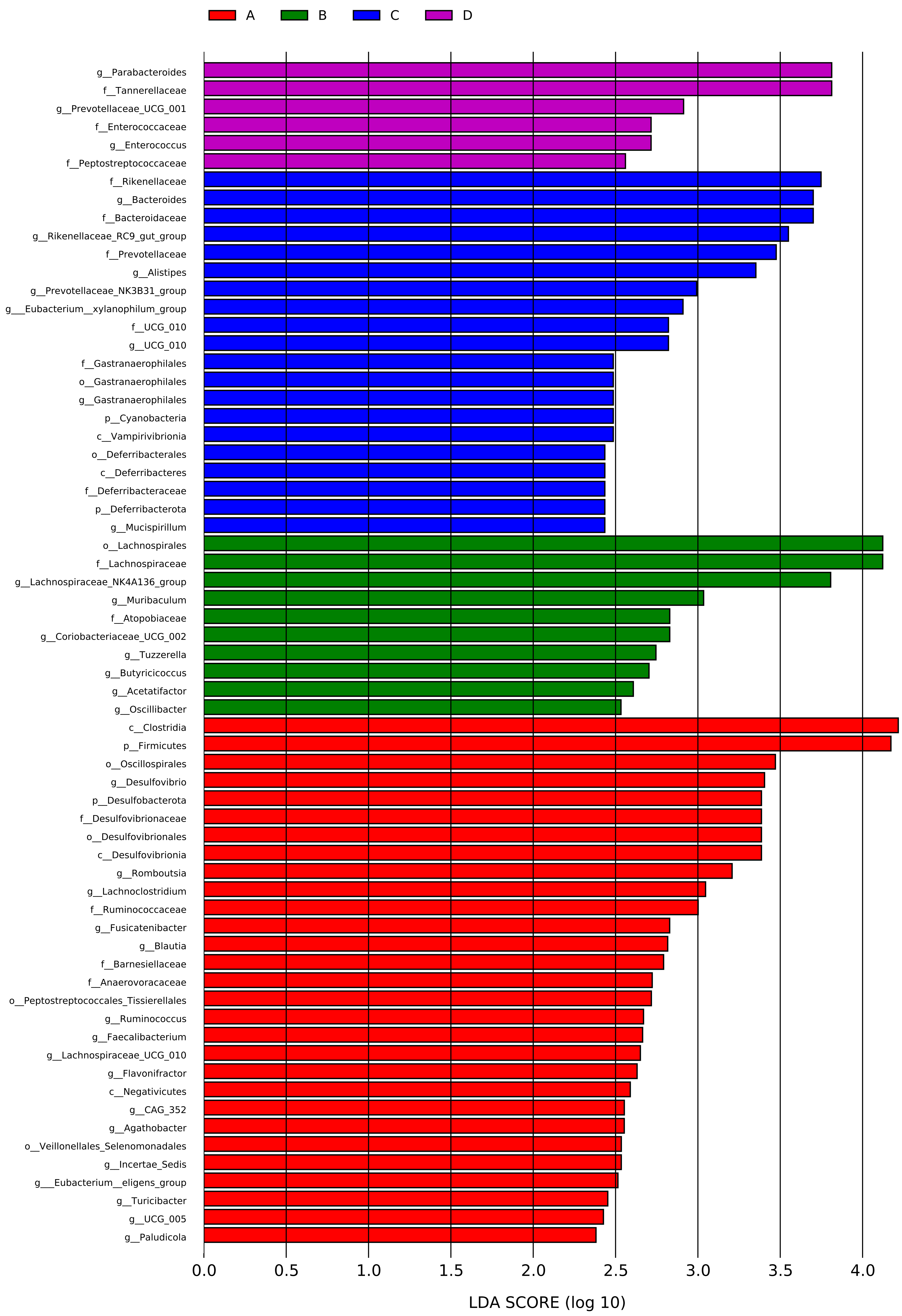

A
B
C
D
g__Parabacteroides
f__Tannerellaceae
g__Prevotellaceae_UCG_001
f__Enterococcaceae
g__Enterococcus
f__Peptostreptococcaceae
f__Rikenellaceae
g__Bacteroides
f__Bacteroidaceae
g__Rikenellaceae_RC9_gut_group
f__Prevotellaceae
g__Alistipes
g__Prevotellaceae_NK3B31_group
g___Eubacterium__xylanophilum_group
f__UCG_010
g__UCG_010
f__Gastranaerophilales
o__Gastranaerophilales
g__Gastranaerophilales
p__Cyanobacteria
c__Vampirivibrionia
o__Deferribacterales
c__Deferribacteres
f__Deferribacteraceae
p__Deferribacterota
g__Mucispirillum
o__Lachnospirales
f__Lachnospiraceae
g__Lachnospiraceae_NK4A136_group
g__Muribaculum
f__Atopobiaceae
g__Coriobacteriaceae_UCG_002
g__Tuzzerella
g__Butyricicoccus
g__Acetatifactor
g__Oscillibacter
c__Clostridia
p__Firmicutes
o__Oscillospirales
g__Desulfovibrio
p__Desulfobacterota
f__Desulfovibrionaceae
o__Desulfovibrionales
c__Desulfovibrionia
g__Romboutsia
g__Lachnoclostridium
f__Ruminococcaceae
g__Fusicatenibacter
g__Blautia
f__Barnesiellaceae
f__Anaerovoracaceae
o__Peptostreptococcales_Tissierellales
g__Ruminococcus
g__Faecalibacterium
g__Lachnospiraceae_UCG_010
g__Flavonifractor
c__Negativicutes
g__CAG_352
g__Agathobacter
o__Veillonellales_Selenomonadales
g__Incertae_Sedis
g___Eubacterium__eligens_group
g__Turicibacter
g__UCG_005
g__Paludicola
0.0
0.5
1.0
1.5
2.0
2.5
3.0
3.5
4.0
LDA SCORE (log 10)
